# Communicating with conscious mechanically ventilated critically ill patients: let them speak with deflated cuff and an in-line speaking valve!

**DOI:** 10.1186/s13054-016-1587-8

**Published:** 2017-01-10

**Authors:** Peter H. Egbers, E. Christiaan Boerma

**Affiliations:** Department of Intensive Care, Medical Centre Leeuwarden, P.O. Box 888, 8901 BK Leeuwarden, Netherlands

With great interest we read the article in *Critical Care* by ten Hoorn et al. [1]. We appreciate the efforts of the authors to write the first review about interventions enabling communication with critically ill patients and to develop an algorithm to select a communication technique. Their attention was focused on patients who were completely ventilator dependent. In cases where tracheotomized patients are able to tolerate cuff deflation, a spontaneous breathing trial with a one-way speaking valve is suggested in their algorithm. However, we would like to point out that this algorithm lacks an important alternative in this particular patient group.

Several studies have been reported on tracheotomized ventilator-dependent critically ill patients who are able to speak with a speaking valve in the respiratory circuit. Speech by tracheotomized ICU patients during mechanical ventilation with a deflated cuff had already been described by Manzano (Verbal communication of ventilator-dependent patients. Crit Care Med 1993;21(4):512-517). In recent years we are aware of at least three articles addressing the issue of enabling speech during weaning of tracheotomized patients off the ventilator. Egbers et al. [2] described their experience with a high-flow ventilator, a deflated cuff, and an in-line speaking valve. Sutt et al. [3] restored speech with use of an in-line speaking valve earlier compared to patients that only used a speaking valve during spontaneous breathing trials. Despite a deflated cuff, lung recruitment improved [4] and in-line speaking valve use is part of their standard care in prolonged weaning. In a randomized clinical trial by Freeman-Sanderson et al. [5] the intervention group received early cuff deflation and insertion of an in-line speaking valve during mechanical ventilation. Restoration of phonation was significantly sooner and without an increase in complications compared to standard therapy with a speaking valve and trials of spontaneous breathing [5].

Although it was partially beyond the scope of the literature search by ten Hoorn et al. (search closed December 2015), we would like the readers of *Critical Care* to draw their attention to this possibility, which has considerable potential to enable speech in tracheotomized patients who are (not yet) able to sustain longer periods of spontaneous breathing trials.
